# Composition and cytotoxic activity of essential oils from *Xylopia aethiopica* (Dunal) A. Rich*, Xylopia parviflora* (A. Rich) Benth.) and *Monodora myristica* (Gaertn) growing in Chad and Cameroon

**DOI:** 10.1186/1472-6882-14-125

**Published:** 2014-04-04

**Authors:** Issakou Bakarnga-Via, Jean Baptiste Hzounda, Patrick Valere Tsouh Fokou, Lauve Rachel Yamthe Tchokouaha, Magali Gary-Bobo, Audrey Gallud, Marcel Garcia, Lucain Walbadet, Youssouf Secka, Pierre Michel Jazet Dongmo, Fabrice Fekam Boyom, Chantal Menut

**Affiliations:** 1 Antimicrobial Agents Unit, Faculty of science, University of Yaoundé I, P.O Box 812, Yaoundé, Cameroon; 2 Department of Biology, Faculty of Science, University of Adam Barka-Abeche, P.O. Box 1173, Abeche, Chad; 3 Faculty of Science, University of Douala, P.O. Box 24157, Douala, Cameroon; 4 IBMM, UMR 5247, Equipe Glyco et nanovecteurs pour le ciblage thérapeutique, Université de Montpellier 1, Faculté de Pharmacie, 15 Avenue Charles Flahault, BP 14491, 34093 Montpellier, France; 5 Institute of Medical Research and Medicinal Plants Studies (IMPM), Yaoundé, Cameroon

**Keywords:** Essential oils, Cytotoxicity, MCF-7 cells, ARPE-19 cells, Monoterpene, Sesquiterpene

## Abstract

**Background:**

Cancer has become a global public health problem and the search for new control measures is urgent. Investigation of plant products such as essential oils from *Monodora myristica*, *Xylopia aethiopica* and *Xylopia parviflora* might lead to new anticancer therapy. In this study, we have investigated the antineoplastic activity of essential oils from fruits of these plants growing in Chad and Cameroon.

**Methods:**

The essential oils obtained by hydrodistillation of fruits of *Monodora myristica*, *Xylopia aethiopica* and *Xylopia parviflora* collected in Chad and Cameroon were analyzed by GC-FID and GC-MS and investigated for their antiproliferative activity against the breast cancer cell line (MCF7).

**Results:**

Overall, monoterpenes were mostly found in the six essential oils. Oils from *X. aethiopica* and *X. parviflora* from Chad and Cameroon mainly contain β-pinene at 24.6%, 28.2%, 35.7% and 32.9% respectively. *Monodora myristica* oils from both origins contain mainly α-phellandrene at 52.7% and 67.1% respectively. The plant origin did not significantly influence the chemical composition of oils. The six essential oils exerted cytotoxic activity against cancer (MCF-7) and normal cell lines (ARPE-19), with more pronounced effect on neoplastic cells in the majority of cases. The highest selectivity was obtained with the essential oils of *X. parviflora* from Chad and Cameroon (5.87 and 5.54) which were more cytotoxic against MCF-7 than against normal cell line (ARPE-19) with IC_50_ values of 0.155 μL/mL and 0.166 μL/mL respectively.

**Conclusions:**

Essential oils from fruits of *Monodora myristica*, *Xylopia aethiopica* and *Xylopia parviflora* have shown acceptable antineoplastic potency, and might be investigated further in this regard.

## Background

Breast cancer is the most frequently diagnosed cancer and the leading cause of cancer death among women, and is the second cause of mortality in sub-Saharan countries [1,2]. According to the GLOBOCAN 2010 estimates, the most common cancer sites in Africa indicate that Cameroon and Chad are highly burdened by breast cancer which is the most frequent cancer in women before the cervical cancer with an incidence rate of 27.9 per 100,000 [3]. These statistics indicate that urgent strategies should be implemented to slowdown the increase of cancer incidence in Africa.

Although there are many therapeutic strategies including chemotherapy, radiation and combination therapies to treat cancer, high systemic toxicity and drug resistance limit the successful outcomes in most cases [4]. Natural products and their synthetic derivatives may be considered as a potential source of novel compounds for the treatment of numerous diseases, comprising over 60% of the approved anticancer drug candidates developed between 1981 and 2002 [5]. Particular attention has been set on the use of essential oils from tropical origin for their cytotoxic activity [6]. Essential oils have shown cytotoxic activity generally without being mutagenic in various organisms [7,8].

The biological properties of aromatic plants such as Annonaceae plants are partially attributed to their essential oils [9]. Aromatic plant such as *Xylopia aethiopica* are used as spices (fruits) all over central and western Africa, and as a component of herbal medicines for the treatment of skin infections, cough, bronchitis, dysentery, female sterility and fever [10,11]. The essential oil of *Xylopia aethiopica* stem bark was investigated by Boyom et al. [12] and showed activity against the W2 strain of *Plasmodium falciparum* in culture. *Monodora myristica* grows naturally in evergreen forests from Liberia to Nigeria and Cameroon, Angola and also Uganda and west Kenya. The fruits and seeds are dried and sold whole or ground to be used in stews, soups, cakes and desserts; they are used as stimulants, stomachic, against headaches, sores and also as insect repellent. In medicine, the bark is used in treatments of stomach-aches, febrile pains, eye diseases and haemorrhoids [13].

Monoterpenes, found in a wide variety of plants including Annonaceae, may be prominent in plant essential oils. In this chemical class, limonene was shown to prevent carcinogen-induced breast cancer at the initiation and the promotion/progression stages. This terpene also causes the complete regression of the majority of advanced rat mammary cancer when added to the diet [14]. In the context of our previous investigations on aromatic plants from tropical areas, the chemical analyses of essential oils obtained by hydrodistillation of fruits of *X. aethiopica, X. parviflora* and *M. myristica* collected in Cameroon showed that all of them were dominated by monoterpenes [15,16]. We therefore hypothesized that these essential oils could show potent cytotoxic activity against human breast cancer cells (MCF-7). This led us to carry out further chemical investigations on the essential oils of their fruits collected in Chad and Cameroon and to evaluate their antiproliferative activity by MTT assay.

## Methods

### Plant material and extraction procedure

The fruits of *Monodora myristica*, *Xylopia aethiopica,* and *Xylopia parviflora* (Annonaceae) were collected from Gore (Chad) and Kribi (Cameroon) in March 2012 following the guidelines for biodiversity exploration and preservation in both countries, filed at the National Herbarium of Yaoundé-Cameroon and at the National Center for Research of Chad, where voucher specimens were identified and deposited. Reference identification numbers for voucher specimens at the National Herbarium in Yaoundé are 27690/SFR/CAM, 28725/SFR/Cam, and 42351HNC respectively for *Monodora myristica*, *Xylopia aethiopica,* and *Xylopia parviflora.*

Essential oils were extracted by hydrodistillation using a Clevenger type apparatus. The fruits were hydrodistilled for 5 h, dried over anhydrous sodium sulfate and then preserved at 4°C free from the light until used. The yields of extraction were calculated in percentage (w/w) relative to the weight of the starting plant material.

### Chemical analysis

The determination of the chemical composition of oils was achieved by gas chromatography and by gas chromatography coupled with mass spectrometry.

### Gas chromatography analysis

Gas chromatography (GC) was performed on a Varian CP-3380 apparatus equipped with flame ionization detector fitted with a fused silica capillary column (30 m × 0.25 mm coated with DB5, film thickness 0.25 μm); temperature program 60-220°C at 3°C/min, injector temperature 200°C, detector temperature 220°C, carrier gas N_2_, 1 mL/min. 0.5 μL of essential oil diluted at 10% in dichloromethane was injected manually. The linear retention indices of the components were determined relatively to the retention times of a series of *n*-alkanes and the percentage compositions were obtained from electronic integration measurements without taking into account relative response factors.

### Gas chromatography-mass spectrometry

GC/MS analyses were performed using a Hewlett–Packard GC 5890 series II equipped with a HP5 (5%-phenyl-methylpolysiloxane) fused silica column (30 m × 0.25 mm; film thickness 0.25 μm) interfaced with a quadrupole detector (Model 5972) applying the same temperature program as for the GC/FID analyses; injector temperature, 220°C; MS transfer line temperature, 250°C; carrier gas, helium at a flow rate of 0.6 mL/min; injection type, split, 1:10 (1 μL 10:100 CH_2_Cl_2_ solution); ionization voltage, 70 eV; electronmultiplier 1460 eV; scan range 35-300 amu; scan rate, 2.96 scan/s. The identification of the constituents was based on comparison of their relative retention indices with either those of authentic samples or with published data in the literature [17] and by matching their mass spectra with those obtained with authentic samples and/or the NBS75K, Wiley 7th NIST 98 EPA/NIH, and FFNSC 2 libraries spectra.

### Evaluation of the cytotoxic activities of essential oils on cancer and normal cell lines

Human breast cancer (MCF-7) and normal epithelial (ARPE-19) cell lines used for cytotoxic activities were purchased from American Type Culture Collection (ATCC, Manassas, VA, USA). They were maintained in Dulbecco’s Modified Eagle Medium (DMEM) F12 (Life Technologies GIBCO, Grand Island, NY, USA) supplemented with 10% foetal bovine serum (FBS) (Sigma-Aldrich St. Louis, MO), glutamine, phenol red and 50 μg/mL gentamycine, in humidified atmosphere at 37°C and 5% CO_2_.

For the cytotoxic study, cells were seeded in 96-well plates at the density of 2,000 cells/well in 200 μL of culture medium and allowed to grow for 24 h. Then serially diluted concentrations of essential oils in 10% absolute ethanol were added to the cells culture at final concentrations ranging from 0.1 to 2 μL/mL and were incubated at 37°C for 72 h. Each concentration was tested in triplicate.

The cell viability was measured by using MTT assay as previously described [18]. The assay detects the reduction of MTT [3-(4,5-dimethylthiazolyl)-2,5-diphenyl-tetrazolium bromide] by mitochondrial dehydrogenase to blue formazan product, which reflects the normal functioning of mitochondrial and cell viability [19]. After 72 h incubation of cells with essential oils, 20 μL of MTT reagent at 0.5 mg/mL was added to each well and incubated for additional 4 h. Then, the medium was removed and 150 μL EtOH/DMSO (1:1) was added to MTT precipitates in each well to solubilize the formazan crystals. The plates were read for optical density at 540 nm, using a microplate reader (Multiskan). Percent inhibition of MCF-7 and ARPE-19 cells was calculated using optical density. The value of cell cytotoxicity at 50% (IC_50_) in the MTT assay was defined as the concentration of test oil resulting in a 50% reduction of absorbance compared with untreated cells. Selectivity indices were calculated for individual oil from the IC_50_ values against normal (ARPE-19) and breast cancer (MCF-7) cell lines as SI = IC_50_ARPE-19/IC_50_MCF-7. Safer essential oils were considered as those with SI > 1.5.

## Results and discussion

### Yields of essential oils extraction

The hydrodistillation of fruits afforded essential oils with 3.57% and 4.68% yields for *X. aethiopica* from Chad and Cameroon respectively. These results are comparable to those previously obtained by Lamaty et al. [15]. The fruits of *X. parviflora* from Chad and Cameroon afforded essential oils with much lower yields (0.76% and 0.68% respectively), close to previous description [16]. Finally, the hydrodistillation of fruits of *M. myristica* collected in Chad and Cameroon gave essential oils with yields of 1.87% and 2.72%, respectively. These yields are lower than those already observed [15]. These yield variations could be inherent to many factors such as site and period of plant collection.

### Chemical composition of the essential oils

The chemical compositions of the essential oil samples are shown in Table 1. All of them contain a majority of monoterpenes (75.0-94.2%) with nevertheless important qualitative and quantitative variations according to the botanical species, while no significant difference was observed according to the geographical origin.

**Table 1:** **Relative percentages of constituents of essential oils from fruits of *X. aethiopica*, *X. parviflora *** **and *M. myristica***

**Components**		***X. aethiopica***		***X. parviflora***		***M. myristica***		**Identification methods**
**LRI**		Chad	CMR	Chad	CMR	Chad	CMR	
**Monoterpenes**		**85.7**	**86.4**	**75.9**	**75.0**	**94.2**	**88.4**	
**Monoterpenes hydrocarbons**		**72.4**	**64.8**	**58.5**	**60.7**	**87.8**	**82.3**	
**926**	α-thujene	1.3	1.0	-	-	2.4	1.4	LRI. GCMS
**931**	α-pinene	8.3	10.8	11.1	10.8	6.7	4.2	LRI. GCMS
**971**	sabinene	14.5	4.8	3.2	3.0	0.3	0.1	LRI. GCMS
**977**	β-pinene	24.6	28.2	35.7	32.9	0.5	0.3	LRI. GCMS
**985**	myrcene	0.3	0.3	-	-	5.1	3.8	LRI. GCMS
**1002**	α-phellandrene	0.6	0.5	-	-	52.7	67.1	LRI. GCMS
**1014**	α-terpinene	2.8	3.4	-	-	0.2	0.1	LRI. GCMS
**1022**	p-cymene	0.6	0.8	1.2	2.8	tr	tr	LRI. GCMS
**1028**	limonene	1.7	0.4	0.3	0.5	14.9	1.8	LRI. GCMS
**1029**	β-phellandrene	10.4	5.8	0.6	0.7	4.2	3.0	LRI. GCMS
**1030**	1,8-cineole	-	-	1.0	2.0	**-**	-	LRI. GCMS
**1032**	(*Z*)-β-ocimene	1.1	1.3	tr	Tr	0.3	0.3	LRI. GCMS
**1042**	(*E*)-β-ocimene	0.2	1.3	5.4	8.0	0.1	0.2	LRI. GCMS
**1055**	γ-terpinene	4.9	5.7	-	-	0.4	tr	LRI. GCMS
**1085**	terpinolene	1.1	1.5	-	-	tr	tr	LRI. GCMS
**Oxygenated monoterpenes**		**13.3**	**21.6**	**17.4**	**14.3**	**6.4**	**3.4**	
**1095**	*trans*-sabinene hydrate	0.4	0.3	-	-	**-**	**-**	LRI. GCMS
**1096**	linalool	-	-	0.6	0.7	2.4	2.1	LRI. GCMS
**1119**	*cis*-p-menth-2-en-1-ol	0.3	0.3	-	-	0.2	tr	LRI. GCMS
**1136**	*trans*-p-menth-2-en-1-ol	-	-	-	-	0.1	tr	LRI. GCMS
**1138**	*trans-*pinocarveol	0.6	0.9	4.0	3.2	**-**	**-**	LRI. GCMS
**1144**	*trans*-verbenol	-	-	0.7	0.4	**-**	**-**	LRI. GCMS
**1163**	pinocarvone	-	-	1.2	0.7	**-**	**-**	LRI. GCMS
**1166**	borneol	-	-	0.6	0.6	**-**	**-**	LRI. GCMS
**1178**	terpinen-4-ol	10.0	15.1	0.8	0.7	**-**	**-**	LRI. GCMS
**1187**	p-cymen-8-ol	-	-	1.0	0.7	-	-	LRI. GCMS
**1189**	α-terpineol	1.6	3.6	1.4	1.7	0.5	0.6	LRI. GCMS
**1193**	myrtenol	-	-	6.5	5.2	**-**	**-**	LRI. GCMS
**1195**	mytenal	0.4	1.4	-	-	**-**	**-**	LRI. GCMS
**1195**	*trans*-thujen-3-ol	-	-	-	-	2.8	0.7	LRI. GCMS
**1234**	*cis*-thujen-3-ol	-	-	**-**	**-**	0.1	-	LRI. GCMS
**1285**	bornyl acetate	-	-	0.6	0.4	**-**	**-**	LRI. GCMS
**1295**	thymol	-	-	**-**	**-**	0.3	-	LRI. GCMS
**Sesquiterpenes**		**12.6**	**11.6**	**23.1**	**23.6**	**3.3**	**11.2**	
**Sesquiterpene hydrocarbons**		**12.0**	**10.2**	**10.3**	**10.3**	**1.9**	**6.7**	
**1337**	δ-elemene	0.9	0.8	tr	Tr	**-**	**-**	LRI. GCMS
**1340**	α-cubebene	0.2	0.4	tr	Tr	**-**	**-**	LRI. GCMS
**1369**	α-ylangene	0.8	0.4	0.4	0.6	**-**	**-**	LRI. GCMS
**1377**	α-copaene	tr	tr	0.4	0.5	**-**	**-**	LRI. GCMS
**1391**	β-elemene	0.4	0.5	0.6	0.4	0.1	0.1	LRI. GCMS
**1422**	β-caryophyllene	0.3	tr	tr	Tr	0.2	0.3	LRI. GCMS
**1435**	β-copaene	-	-	1.4	1.1	-	-	
**1450**	α-humulene	0.2	0.8	**-**	**-**	0.1	0.3	LRI. GCMS
**1478**	γ-muurolene	0.4	1.1	0.3	0.5	0.1	0.4	LRI. GCMS
**1485**	germacrene D	7.2	5.1	1.3	2.0	**-**	**-**	LRI. GCMS
**1493**	*trans*-muurola-4(14),5-diène	-	-	1.6	1.8	0.4	0.3	LRI. GCMS
**1499**	bicyclogermacrene	0.5	0.3	**-**	**-**	0.1	-	LRI. GCMS
**1502**	α-muurolene	-	-	0.7	0.8	0.1	0.4	LRI. GCMS
**1515**	γ-cadinene	-	-	**-**	**-**	0.1	1.7	LRI. GCMS
**1523**	δ-cadinene	0.7	0.3	3.6	3.7	0.7	3.2	LRI. GCMS
**1561**	germacrene B	0.4	0.5	**-**	**-**	**-**	**-**	LRI. GCMS
**Oxygenated sesquiterpenes**		**0.6**	**1.4**	**12.8**	**13.3**	**1.4**	**4.5**	
**1518**	cubebol	-	-	1.5	1.7	-	-	LRI. GCMS
**1548**	elemol	-	-	1.5	2.0	-	**-**	LRI. GCMS
**1578**	germacrene D-4-ol	-	-	**-**	**-**	0.5	1.3	LRI. GCMS
**1589**	caryophyllene oxide	-	-	3.0	2.0	**-**	**-**	LRI. GCMS
**1630**	M = 220	0.3	1.1	1.0	0.8	**-**	**-**	GCMS
**1642**	*epi*-α-cadinol	-	-	2.0	2.4	0.1	0.9	LRI. GCMS
**1647**	α-muurolol	-	-	2.0	2.9	**-**	**-**	LRI. GCMS
**1652**	α-cadinol	0.3	0.3	**-**	**-**	0.1	0.9	LRI. GCMS
**1686**	germacra-4(15),5, 10(14)-triene-1- α-ol	-	-	1.8	1.5	-	-	LRI. GCMS
**1696**	shyobunol	-	-	**-**	**-**	0.7	0.3	LRI. GCMS
**1698**	(*2Z*,*6Z*)-farnesol	-	-	**-**	**-**	-	1.1	LRI. GCMS
**Total identified**		**98.3**	**98.0**	**99.0**	**98.6**	**97.5**	**96.9**	
**EO extraction yields (%)**		**3.57**	**4.8**	**0.7**	**0.68**	**1.87**	**2.7**	

CMR = Cameroon; LRI = linear retention indices on a HP-5 column; - = not found, tr = trace (relative concentration < 0.1%); EO = essential oil.

The essential oils of *X. aethiopica* from Chad and Cameroon were rich in monoterpene hydrocarbons with contents of 72.4% and 64.8% respectively. The main constituents were β-pinene (24.6.9-28.2%), sabinene (4.8-14.5%), β-phellandrene (5.8-10.4%) and γ-terpinene (4.9-5.7%). Oxygeneted monoterpenes were significantly represented (13.3-21.6%) dominated by terpinen-4-ol (10.0-15.1%). These results corroborate those obtained by Lamaty et al. [15,16] as well as those reported more recently by Keita et al. [20] and Noudjou et al. [21]. Of note, the stem bark oil previously investigated by Boyom et al. [12] was qualitatively distinct with β-pinene (10.07%), myrtenol (6.4%), spathulenol (6.33%), and γ-ylangene (5.32%) as major constituents.

The essential oils obtained by hydrodistillation ot fruits of *X. parviflora* collected in Chad and Cameroon were also dominated by pinenes (43.7 to 46.8), but they differ from the samples of *X. aethiopica* oils by a minor content of sabinene (3.0 to 3.2%) and β-phellandrene (0.6 to 0.7%), while (*E*)-β-ocimene accounted for 5.4 to 8.0% of the volatile extracts. They were also characterized by high content of sesquiterpenes (23.1 to 23.6%), dominated by cadinene and muurolane skeletons (i. e. δ-cadinene, α-and γ-muurolenes, *trans*-muurola-4(14),5-diene, *epi*-α-cadinol, α-muurolol) representing 10.2% and 12.1% of the whole oils from Chad and Cameroon respectively. These results corroborate those obtained by Lamaty et al. [16] from essential oil of fruits of *X. parviflora* collected in the Bayagam area (Cameroon).

The essential oils obtained from fruits of *M. myristica* collected in Chad and Cameroon predominantly consisted of monoterpene hydrocarbons (87.8% and 82.3% respectively), the oxygenated derivatives representing only 6.4% and 3.4% of the whole oils. Both samples contained α-phellandrene as major compound with 52.7% and 67.1% respectively. The sample from Chad is characterized by a high content of limonene (14.9%); on the other hand the oil from Cameroon differs by more than 10% of sesquiterpenes (mainly cadinane derivatives). This result is comparable to that of Lamaty et al. [15] who found 48.8% of α-phellandrene in a sample collected in Yaoundé area (Cameroon).

### Cell cytotoxicity of essential oils

The cytotoxic activity of all essential oils was evaluated on human breast cancer (MCF-7) and normal epithelial (ARPE-19) cell lines using the MTT assay based on cell viability. Cells were exposed to the oils at concentrations ranging from 0.1 to 2 μL/mL.

**Figure 1:**
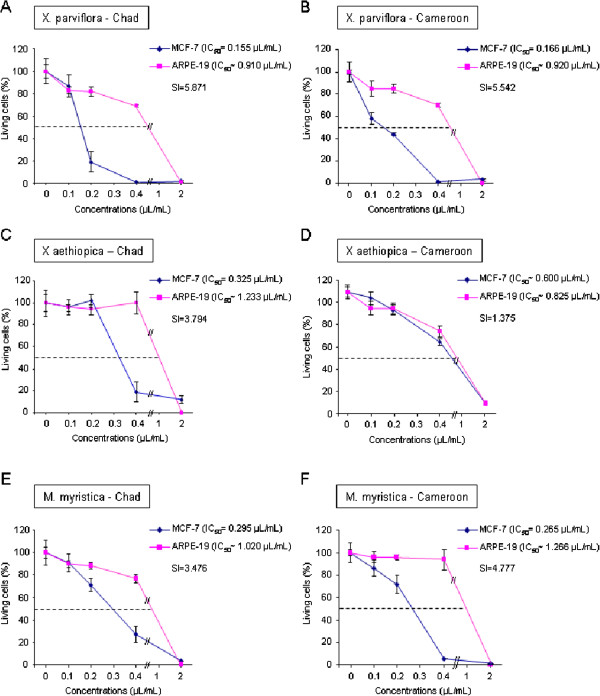
**Dose-dependent cytotoxic effect of the essential oils of X. parviflora from Chad (A) and Cameroon (B), X. aethiopica from Chad (C) and Cameroon (D) and M. myristica from Chad (E) and Cameroon (F).** Human breast cancer (MCF-7) and normal (ARPE-19) cell lines were incubated 72 h with increasing doses of essential oils from 0.1 to 2 μL/mL. After treatment, cytotoxicity was measured by MTT assay as described in Material and Methods. Values are means ± standard deviations of three independent experiments.

The cytotoxic effects of the essential oils on human normal or cancer cell lines are shown in Figure 1. The IC_50_ values and the selectivity indices were given in each case. The essential oils from fruits of *X. aethiopica*, *X. parviflora* and *M. myristica* produced a highly significant (p < 0.005) decrease in living cancer cells (MCF-7) after 72 h of incubation. Importantly, the effects on normal epithelial cells (ARPE-19) were mainly less pronounced.

The fruit essential oils of *X. parviflora* from Chad and Cameroon were the most active on the cancer MCF-7 cells with IC_50_values of 0.155 and 0.166 μL/mL while on normal ARPE-19 cells they showed IC_50_ values of 0.910 and 0.920 μL/mL, expressing a selective cytotoxic effect (SI = 5.871 and 5.542 respectively). These oils showed the best profile as potential sources for cancer therapy development. On the other hand, *X. aethiopica* oils from Chad and Cameroon were less cytotoxic and they exhibited different activity according to their origin. Only the essential oil of *X. aethiopica* from Chad showed a higher cytotoxic effect on cancer cells than on normal cells with a SI = 3.794. The same essential oil coming from Cameroon presented comparable effects on neoplastic and normal cells.

Finally, *M. myristica* essential oil appeared also to be more cytotoxic on cancer cells than on normal cells with a SI = 3.476 for the oil from Chad and SI = 4.777 for the oil from Cameroon.

Many reports have been published on the cytotoxic effect of different Annonaceae plant extracts on human cancer and non-cancer cell lines, but few is known about the cytotoxic effect of the essential oils of *X. aethiopica*, *X. parviflora,* and *M. myristica*. Choumessi et al. [22] and Kuete et al. [23] reported the antiproliferative activity of hydroethanolic extract of *X. aethiopica* against cancer cell lines with IC_50_values of 12 μg/mL against HCT116 colon cancer cells, 7.5 μg/mL and >25 μg/mL against U937 and KG1a leukemia cells, 6.86 μg/mL and 3.91 μg/mL against MiaPaCa-2 and CCRF-CEM cells. Adaramoye et al. [24] also showed the antiproliferative effect of *X. aethiopica* extract on human cervical cancer cells. On the other hand, it was reported that components such as terpinen-4-ol and α-cadinol which are found in all the tested essential oils presented activity against breast, colon, gastric, lung, ovarian and laryngeal cancer cell lines [25]. In addition, β-pinene which is one of the most naturally occurring monoterpene found in the oil of *Xylopia* species, has shown a significant cytotoxic activity against breast cancer and epidermal skin cancer cell lines [26].

Besides, it has been shown that limonene can improve the cytotoxic activity of essential oils against neoplasms through induction of apoptosis and phase 1 and phase 2 carcinogen metabolizing enzymes (cytochrome P450) that metabolize carcinogens to less toxic forms and prevent the interaction of chemical carcinogens with DNA [27]. Though, the *M. myristica* oil from Chad which was rich in limonene (14.9%) did not present the best cytoxic efficiency against MCF-7 cells. The best results obtained for the two *X. parviflora* oils and for the *M. myristica* sample from Cameroon, could be due to their content of oxygenated sesquiterpenes.

Finally, the significant cytotoxic activity against MCF-7 cells of *X. parviflora* fruits oils could be explained by the presence of caryophyllene oxide which was not present in the other samples. Sibanda [28] previously demonstrated the antiproliferative effect of caryophyllene oxide against SK-MEL-28, MDA-MB-231, Hs 578 T, and 5637 cancer cell lines. They also found that caryophyllene oxide (100 μg/mL) was cytotoxic on breast cancer (MCF-7) and prostate cancer (PC-3) cells with 89.67% and 96.75% of cell death respectively.

Definitely, the high monoterpenic content in *X. aethiopica* and *M. myristica* oils and the occurrence of potent anticancer compounds such as limonene in these extracts did not influence their cytotoxicity against MCF-7 cells, contrary to the findings of Miller et al. [29]. In the contrary, the essential oils from *X. parviflora* lacking this component showed the best activity against the cancer cells. This is an indication that anticancer activity might be rather related to a sum of interactions between oils components [30], or to the occurrence of different or trace elements.

## Conclusion

The results from this work showed that essential oils of *X. aethiopica, X. parviflora* and *M. myristica* fruits from Chad and Cameroon were qualitatively comparable, within the same botanical species with modest quantitative variations. Their higher cytotoxic effect on breast cancer cells (MCF-7) than on normal cells (ARPE-19 cells) highlighted their potential as source of eventual therapy against human breast cancer. Further detailed investigations are required to light on specific components that elicit cytotoxic activity and identify their mechanisms of action.

## Abbreviations

GC/MS: Gas chromatography-mass spectrometry; MTT: [3-(4,5-dimethylthiazolyl)-2,5-diphenyl-tetrazolium bromide]; GC: Gas chromatography; CMR: Cameroon; LRI: Linear retention indices on a HP-5 column; - = not found; Tr: Trace (concentration < 0.1%); EO: Essential oils.

## Competing interests

The authors declare that they have no competing interests.

## Authors’ contributions

FFB, PMJD, IB-V, JBHF, VPFT, LRYT, MGB, and YS designed the study and participated in plant selection and collection, spectral analyses and in the drafting and correction of the manuscript. IB-V, MG, LW, AG, and CM performed GC, GC/SM analyses of the essential oils and carried out the cytotoxicity assay. They also contributed to data analysis and critically revised the manuscript. IB-V, JBHF, VPFT, and LRYT extracted the essential oils. All the authors read and approved the final manuscript version.

## Pre-publication history

The pre-publication history for this paper can be accessed here:

http://www.biomedcentral.com/1472-6882/14/125/prepub
